# Development of Three-Dimensional Human Intestinal Organoids as a Physiologically Relevant Model for Characterizing the Viral Replication Kinetics and Antiviral Susceptibility of Enteroviruses

**DOI:** 10.3390/biomedicines9010088

**Published:** 2021-01-18

**Authors:** Jessica Oi-Ling Tsang, Jie Zhou, Xiaoyu Zhao, Cun Li, Zijiao Zou, Feifei Yin, Shuofeng Yuan, Man-Lung Yeung, Hin Chu, Jasper Fuk-Woo Chan

**Affiliations:** 1 State Key Laboratory of Emerging Infectious Diseases, Carol Yu Centre for Infection, Department of Microbiology, Li Ka Shing Faculty of Medicine, The University of Hong Kong, Hong Kong, China; joltsang@hku.hk (J.O.-L.T.); jiezhou@hku.hk (J.Z.); xyzhao89@connect.hku.hk (X.Z.); licun@hku.hk (C.L.); u3006001@connect.hku.hk (Z.Z.); yuansf@hku.hk (S.Y.); pmlyeung@hku.hk (M.-L.Y.); hinchu@hku.hk (H.C.); 2 Hainan-Medical University-The University of Hong Kong Joint Laboratory of Tropical Infectious Diseases, The University of Hong Kong, Pokfulam, Hong Kong, China; yinfeifei@hainmc.edu.cn

**Keywords:** antiviral, enterovirus, intestinal, organoids, platform

## Abstract

Enteroviruses are important causes of hand, foot, and mouth disease, respiratory infections, and neurological infections in human. A major hurdle for the development of anti-enterovirus agents is the lack of physiologically relevant evaluation platforms that closely correlate with the in vivo state. We established the human small intestinal organoids as a novel platform for characterizing the viral replication kinetics and evaluating candidate antivirals for enteroviruses. The organoids supported productive replication of enterovirus (EV)-A71, coxsackievirus B2, and poliovirus type 3, as evidenced by increasing viral loads, infectious virus titers, and the presence of cytopathic effects. In contrast, EV-D68, which mainly causes respiratory tract infection in humans, did not replicate significantly in the organoids. The differential expression profiles of the receptors for these enteroviruses correlated with their replication kinetics. Using itraconazole as control, we showed that the results of various antiviral assays, including viral load reduction, plaque reduction, and cytopathic effect inhibition assays, were highly reproducible in the organoids. Moreover, itraconazole attenuated virus-induced inflammatory response in the organoids, which helped to explain its antiviral effects and mechanism. Collectively, these data showed that the human small intestinal organoids may serve as a robust platform for investigating the pathogenesis and evaluating antivirals for enteroviruses.

## 1. Introduction

Enteroviruses are small, non-enveloped RNA viruses belonging to the genus *Enterovirus* in the family *Picornaviridae* that are associated with major human and mammalian diseases. There are 15 species in the genus *Enterovirus* [1]. Most of the human-pathogenic enteroviruses belong to the species *Enterovirus* A to D or *Rhinovirus* A to C. Humans mainly acquire enteroviruses through the fecal-oral route and, sometimes, via the respiratory tract. After ingestion, enteroviruses replicate in the pharynx and small intestinal submucosal lymphoid tissues and mature polarized enterocytes [2]. Then, enteroviruses pass to regional lymph nodes and cause transient viremia to infect reticuloendothelial tissues including liver, spleen, and bone marrow. In a minority of patients, disseminated infection with tissue necrosis and inflammation involving the central nervous system, heart, and skin may occur. In particular, Enterovirus A71 (EV-A71), a major cause of hand, foot, and mouth disease, especially among children in the Asia-Pacific region, may be associated with severe systemic complications including meningoencephalitis, myelitis, carditis, and non-cardiogenic pulmonary edema with respiratory failure and even death [3,4]. 

Despite its clinical importance, antiviral options remain lacking for EV-A71 infection. The antiviral development for EV-A71 infection has been hampered partly by the lack of physiologically relevant in vitro models as well as readily available animal models. Traditionally, a number of cancer cell lines, such as rhabdomyosarcoma (RD) and colonic adenocarcinoma (Caco-2) cells, have been used for testing candidate anti-EV-A71 agents. However, the results obtained from these non-physiological cell lines may be suboptimally translational. In vivo antiviral evaluation usually requires the use of suckling mice or mouse-adapted virus strains which are not readily available in many laboratories. Thus, the establishment of a physiologically relevant model suitable for evaluating potential anti-enteroviral agents is in urgent need.

The advances in organoid development in recent years have allowed the use of human small intestinal organoids for modeling infections of different enteric viruses such as human rotavirus and norovirus [5,6,7]. The human small intestine is a complex organ tissue with a polarized epithelial layer that contains different cell types to perform various critical functions such as secretion of mucus and defensins, and the absorption of water and nutrients. The human small intestinal organoid is a three-dimensional (3D) spheroid consisting of a stem cell niche and all the differentiated epithelial cell types surrounding a single luminal compartment [8]. The differentiated epithelial cells also retain the characteristic apical-basal polarity which has major impact on virus entry and dissemination [9,10]. Importantly, the human small intestinal organoids contain the major absorptive (enterocytes) and secretory (Paneth, goblet, and enteroendocrine) cell types of the intestinal epithelium, thereby highly resembling most functional properties of the small intestine in vivo [11]. Recently, this state-of-the-art model has also been applied for studying enteroviruses [11,12]. In this study, we first characterized the viral replication kinetics of the human-pathogenic species A to D enteroviruses in the human small intestinal organoid model. We then compared the performance of human small intestinal organoid model with that of traditional cell lines for evaluation of itraconazole (ITZ), a clinically approved antifungal agent with documented anti-enteroviral activity [13,14,15]. We further assessed the mRNA transcript expressions of selected host immunity genes and chemokines/cytokines after the drug treatment in the infected small intestinal organoids. Our results showed that the human small intestinal organoid model is a physiologically relevant model that supports the productive replication of various enteroviruses and may serve as a reliable platform for evaluating candidate anti-enteroviral agents.

## 2. Materials and Methods

### 2.1. Cell Culture, Human 3D Small Intestinal Organoids and Drug Compound 

RD (ATCC, CCL-136, Manassas, VA, USA), Caco-2 (ATCC, HTB-37, Manassas, VA, USA), Vero (ATCC, CCL-81, Manassas, VA, USA) cells were maintained in Dulbecco’s modified Eagle’s medium (DMEM) (Gibco, Waltham, MA, USA) supplemented with 10% fetal bovine serum (FBS) (Gibco). The cell cultures were incubated at 37 ℃ under 5% CO_2_. Human 3D small intestinal organoids were generated based on the previous protocol [16]. Ethical approval was obtained from the Institutional Review Board of the University of Hong Kong/Hospital Authority Hong Kong West Cluster for the use of human tissues. Briefly, small pieces of small intestinal tract tissues were obtained from patients who provided informed consent during operative procedures of the gastrointestinal tract at Queen Mary Hospital, Hong Kong. The tissues were minced and dissociated with 10 mM EDTA (Sigma-Aldrich, St. Louis, MO, USA) for 1 h at 4 °C, followed by pipetting up and down. The crypt fractions were then collected by centrifugation and embedded in thawed Matrigel (Corning, Corning, NY, USA). The generated small intestinal organoids were maintained in culture medium (CM) (Appendix A, Table A1) at 37 °C under 5% CO_2_. The differentiated organoids were used for infection after cultivation in CM for 5 days and differentiation for 4 days in differentiation medium. The differentiation medium consisted of the same components as found in the culture medium but without the addition of Wnt3A, SB202190, and nicotinamide, and with halved concentrations of Noggin and R-spondin-1. To quantify the cell number of organoids for calculation of multiplicity of infection (MOI), the organoids were dissociated into single cell suspensions using 10× TrypLE Select Enzyme (Gibco for 1–5 min at 37 °C. The cell number was counted using the cell counting chamber (Paul Marienfeld, Lauda-Königshofen, Germany). ITZ (Sigma-Aldrich) was prepared as 10 mM stock in absolute dimethyl sulfoxide (DMSO) (Sigma-Aldrich) and the aliquots were stored at −20 °C until use.

### 2.2. Virus Strains and Titration

Archived clinical isolates of species A to D enteroviruses were available at the Department of Microbiology, The University of Hong Kong. EV-A71, poliovirus type 3 (PV-3), and enterovirus D68 (EV-D68) (Appendix A, Table A2) were propagated in DMEM supplemented with 2% FBS in RD cells while coxsackievirus B2 (CVB2) (Appendix A, Table A2) was propagated in DMEM supplemented with 2% FBS in Vero cells at 37 °C under 5% CO_2_ as previously described [17]. The supernatants were harvested when 80% of the cells exhibited cytopathic effects (CPE) and then centrifuged to produce virus stocks which were immediately stored at −80 °C until use. Titers of all virus stocks were determined by TCID_50_ assay. TCID_50_ assays of EV-A71, CVB2, and PV-3 stocks were performed in RD cells while that of CVB2 stock was performed in Vero cells. Each virus stock was diluted by tenfold serially with four technical replicates.

### 2.3. Virus Infection of Human Small Intestinal Organoids and Cell Lines

For human small intestinal organoid infection, the organoids were washed twice with cold basal medium to remove Matrigel. The organoids were then sheared by vigorously pipetting 20–30 times to expose the apical surface to the virus inoculum. After virus inoculation (MOI = 0.01, 0.05, or 1.00) with EV-A71, CVB2, PV-3, or EV-D68, or mock infection, the mixture was then incubated for 2 h at 37 °C for virus adsorption. After washing for three times, the infected organoids were re-embedded in Matrigel as a droplet and maintained in differentiation medium. At the indicated time points, the culture supernatant, dissolved Matrigel, and pelleted organoids were collected for viral load quantification. For each well, Matrigel of the droplet was dissolved using cell recovery solution (Corning) and the organoids were pelleted after centrifugation at 1000× *g* for 5 min. The culture supernatant and dissolved Matrigel were pooled as a complete supernatant sample. For cell line infection, the cell monolayer was washed once with phosphate-buffered saline (PBS) (Gibco) and then incubated with virus inoculum (MOI = 0.01 in RD cells; MOI = 0.05 in Caco-2 cells) for 1 h at 37 °C for virus adsorption. After washing twice, the monolayer was cultured in DMEM supplemented with 2% FBS. At the indicated time points, the culture supernatant and cell lysate were collected for viral load quantification.

### 2.4. Viral Replication Kinetics

The small intestinal organoids were virus-infected or mock-infected. The time was set as zero before adsorption for 2 h. The supernatant and organoid lysate samples were then harvested at 6 h post-inoculation (hpi), 24 hpi, 48 hpi, and 72 hpi for viral load quantification. In parallel, the cell morphology was monitored and bright-field micrographs were taken at the indicated time points using a Nikon Eclipse TS100 Inverted Routine Microscope associated with a CCD camera and computer (Nikon, Tokyo, Japan). The cell viabilities of each virus-infected and mock-infected group were evaluated at the indicated time points using CellTiter-Glo^®^ 3D cell viability assay (Promega Corporation, Madison, WI, USA). The cell viabilities were calculated as % mock using the formula: (luminescence reading_infected group_/luminescence reading_mock-infected group_) ×100.

### 2.5. Cell Viability and CPE Inhibition Assays

The 50% effective cytotoxic concentrations (CC_50_) of ITZ in RD and Caco-2 cells as well as the drug’s cell protection effects against EV-A71 infection in these cells were determined by CellTiter-Glo^®^ luminescent cell viability assay as previously described (Promega Corporation) [18]. For the human small intestinal organoids, the CC_50_ of ITZ and the drug’s cell protection effects against EV-A71 infection were determined by the CellTiter-Glo^®^ 3D cell viability assay (Promega Corporation) according to the manufacturer’s instructions. 

For determination of ITZ’s CC_50_, the non-infected cells and organoids were treated with different concentrations of ITZ (0–50 μM) for 48 h. The CC_50_ was calculated using non-linear regression (curve fit) with GraphPad Prism 5.0 software (GraphPad, San Diego, CA, USA). For cell protection effect, the cells or organoids were first infected by EV-A71 and incubated for 1 h (RD and Caco-2 cell lines) to 2 h (organoids). Then, the infected cells or organoids were treated with DMSO (i.e., 0 μM ITZ) or up to 3 μM ITZ for 48 h. After 48 h, the CellTiter-Glo^®^ reagent was added to the cells or organoids according to the manufacturer’s instructions. After shaking and signal stabilization for 30 min, the luminescence signal was detected by the Victor X3 2030 Multilabel Reader (PerkinElmer, Waltham, MA, USA) according to the manufacturer’s instructions. The % relative survivability was calculated as follows: (luminescence reading_ITZ treated, infected_—luminescence reading_DMSO control, infected_)/(luminescence reading_DMSO control, uninfected_—luminescence reading_DMSO control, infected_) ×100.

### 2.6. Viral Load Reduction and Plaque Reduction Assays

Viral load reduction assay was performed to evaluate the antiviral activity of ITZ against EV-A71 infection as previously described with modifications [19,20,21,22]. Briefly, EV-A71-infected RD cells (MOI = 0.01), Caco-2 cells (MOI = 0.05), and organoids (MOI = 0.05) were treated with different concentrations of ITZ or 0.5% DMSO. Supernatant and cell or organoid lysate samples were collected at 48 hpi for RNA extraction and quantitative reverse transcription-PCR (qRT-PCR) using the primers and probes listed in Table A3 [23]. Additionally, the supernatants were also tested for virus titration by plaque assay as previously described with modifications [24]. Briefly, confluent RD cells were incubated with serially-diluted supernatant at 37 °C for 1 h and subsequently overlaid with 1% low-melting-point agarose (Promega Corporation) after aspirating the inoculum. The cells were fixed with 4% formaldehyde (Sigma-Aldrich) after the plaques were observed and then stained with 0.5% crystal violet. The half maximal inhibitory concentration (IC_50_) was calculated using non-linear regression (curve fit) with GraphPad Prism 5.0 software (GraphPad). The % plaque reduction was calculated as follows: (Plaque number_untreated group_—Plaque number_ITZ treated group_)/Plaque number_untreated group_ × 100.

### 2.7. Quantitative RT-PCR (qRT-PCR)

After the supernatant and cell or organoid lysate samples were harvested, RNA was extracted using RNeasy Plus Mini Kit (Qiagen, Hilden, Germany). The EV viral loads in the samples were detected with the PanEV primers (Appendix A, Table A3) using QuantiNova Probe RT-PCR kit (Qiagen) in a LightCycler^®^ 96 Real-Time PCR System (Roche, Basel, Switzerland) as previously described [25]. The viral gene copy number was determined by absolute quantification using a plasmid expressing the conserved 5′-untranslated region (UTR) among EVs. GAPDH mRNA expression level was detected with specific primers (Appendix A, Table A3) and used as endogenous control for data normalization between lysate samples.

To quantify the mRNA expression levels of EV receptors [26,27,28,29,30,31,32], non-infected RD cells, Caco-2 cells, and organoids were harvested with an approximate cell number of 1 × 10^5^ and then lysed for RNA extraction. To profile the inflammatory and antiviral responses of the EV-A71-infected human small intestinal organoids before and after ITZ treatment, the organoids were first infected with EV-A71 (MOI = 0.05) or mock-infected. Then, the EV-A71-infected samples were treated with either 3 μM ITZ or 0.5% DMSO, and the mock-infected samples were treated with 0.5% DMSO for 48 h. Finally, the harvested samples were lysed for RNA extraction using RNeasy Plus Mini Kit (Qiagen). cDNA was synthesized with Oligo-dT primer using the Transcriptor First Strand cDNA Synthesis Kit according to the manufacturer’s instructions (Roche). qPCR was performed with gene-specific primers (Appendix A, Table A3) [11] using LightCycler^®^ 480 SYBR Green I Master Mix (Roche) in a LightCycler^®^ 96 Real-Time PCR System (Roche). Each 20-μL reaction mixture contained 10 μL of Master Mix, 3 μL of RNase-free water, 1 μL each of 10 μM gene-specific forward and reverse primer, and 5 μL of cDNA as template. Reactions were incubated at 95 °C for 5 min for pre-incubation, followed by 45 amplification cycles of 95 °C for 10 s, 55 °C for 10 s and 72 °C for 10 s. Signal detection and measurements were taken in each amplification cycle. The cycling profile ended with a cooling step at 40 °C for 10 s.

### 2.8. Immunofluorescence Microscopy 

The human small intestinal organoids were washed with 2% FBS/PBS and fixed with 4% paraformaldehyde (Sigma-Aldrich) at room temperature, followed by 0.5% Triton X-100 (Sigma-Aldrich) to permeabilize the cell membranes. The organoids were then blocked with Protein Block (Dako, Santa Clara, CA, USA) for 1 h and incubated with primary Pan-EV antibody from the Light Diagnostics^TM^ Pan-Enterovirus reagent kit (Merck Millipore, Burlington, MA, USA) at 4 °C overnight. After washing, the organoids were incubated with Alexa Fluor^®^488-conjugated secondary antibody (Abcam, Cambridge, UK) at room temperature for 2 h. Nuclei and actin filaments were counterstained with DAPI (Invitrogen, Carlsbad, CA, USA) and Phalloidin-647 (Sigma-Aldrich), respectively. The confocal micrographs were taken using a Carl Zeiss LSM 800 confocal microscope (Zeiss, Oberkochen, Germany).

### 2.9. Statistical Analysis 

All data were analyzed with GraphPad Prism 5.0 software (GraphPad). Student’s *t*-test was used for data analysis. *p* < 0.05 was considered statistically significant. * indicates *p* < 0.05; ** indicates *p* < 0.01; and *** indicates *p* < 0.001.

## 3. Results

### 3.1. Assessment of the Susceptibility of Human Small Intestinal Organoids to Different Species of Enteroviruses by CPE and Cell Viability Assays 

**Figure 1:**
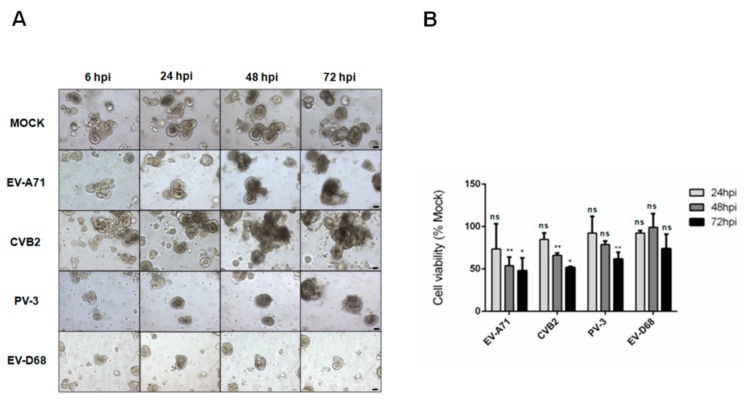
Cytopathic effects and cell viability reduction after infections of human small intestinal organoids with Enterovirus A71 (EV-A71), coxsackievirus B2 (CVB2), poliovirus type 3 (PV-3), or EV-D68. (**A**) Human small intestinal organoids infected at with EV-A71, CVB2, PV-3 or EV-D68 (multiplicity of infection (MOI) = 0.01), or mock-infected control. Representative phase contrast micrographs were taken at 100× magnification (scale bar: 40 μm); (**B**) Cell viability assay of organoids infected with EV-A71, CVB2, PV-3, or EV-D68 (MOI = 0.01), or mock-infected control. Cell viability was measured at 24 hpi, 48 hpi, and 72 hpi using 3D CellTiter-Glo^®^ cell viability assay. Data were shown as mean ± SD (in triplicates). The *p*-values represented the comparison between each individual virus-infected sample vs. mock-infected sample at the indicated time points (Student’s *t* test). * indicates *p* < 0.05; ** indicates *p* < 0.01; ns indicates not significant.

To investigate the susceptibility of human small intestinal organoids to infections by human-pathogenic enteroviruses belonging to different species, we infected the organoids with EV-A71 (species A), CVB2 (species B), PV-3 (species C), and EV-D68 (species D) with a MOI of 0.01. Distinctive CPEs were observed at 48hpi in the EV-A71- or CVB2-infected organoids (Figure 1A). Initially, cell detachment from the surface of the organoids were observed, which progressed to severe cell death with collapsed structures as the infection progressed. Similar progressive CPE changes were also observed in the organoids infected with PV-3 over 72 hpi. In contrast, no CPE was observed in the EV-D68-infected or mock-infected organoids with preserved crypt morphology and integrity for up to 72 hpi. Next, we utilized the cell viability assay to quantify virus-induced cell death. Consistent with the microscopic observations, we showed that the cell viability started to drop at 24 hpi and then progressively decreased to ~50% at 48 hpi in the organoids infected with either EV-A71 or CVB2 (Figure 1B). PV-3-infected organoids demonstrated a similar but slower reduction in cell viability over 72 hpi, whereas EV-D68-infected organoids did not cause significant (*p* > 0.05) reduction in cell viability throughout the 72-h incubation period. Collectively, these results demonstrated that human small intestinal organoids were susceptible to infections by EV-A71, CVB2, and PV-3, but not EV-D68.

### 3.2. Viral Replication Kinetics, Infectious Virus Titers, and Viral Antigen Expression of EV-A71, CVB2, PV-3 and EV-D68 Infections in Human Small Intestinal Organoids

**Figure 2:**
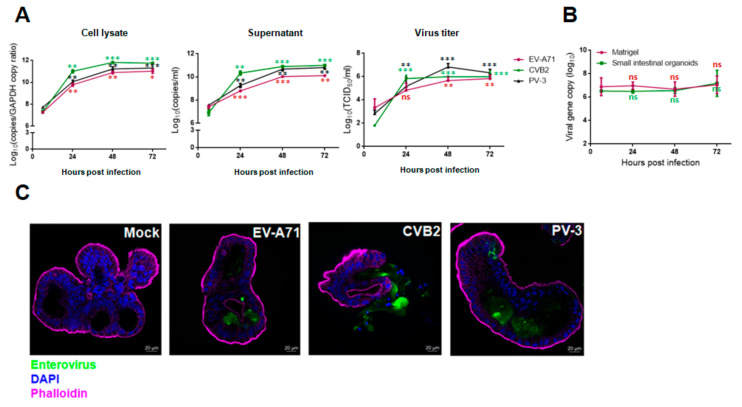
Viral replication kinetics and immunofluorescence staining of human small intestinal organoids infected with EV-A71, CVB2, PV-3, or EV-D68. (**A**) Differential viral replication kinetics of EV-A71, CVB2, or PV-3 in human small intestinal organoids. The organoids were infected with each enterovirus (MOI = 0.01). (**B**) Human small intestinal organoids infected with EV-D68 at high MOI (MOI = 1.00). The data are shown as mean ± SD and in triplicates in (**A**,**B**). (**C**) Human small intestinal organoids infected with EV-A71, CVB2, or PV-3 (MOI = 0.01), or mock-infected, were immunostained for enterovirus antigen (in green) using the Light Diagnostics^TM^ Pan-Enterovirus reagent kit. Organoids were infected with each enterovirus (MOI = 0.01) for 48 h. DAPI-stained nuclei were shown in blue. Phalloidin-stained cytoskeletons were shown in cyan and purple (Scale bar: 20 μm). ** indicates *p* < 0.01; *** indicates *p* < 0.001; ns indicates not significant.

Next, we used qRT-PCR and TCID_50_ assays to quantify the replication kinetics of enteroviruses in human small intestinal organoids. As shown in Figure 2A, EV-A71, CVB2, and PV-3 exhibited similar replication kinetics in the organoids throughout the 72-h incubation period, with the exponential phase occurring between 6 hpi and 48 hpi and the peak viral load being achieved at 72 hpi in the infected organoids as well as the culture supernatant and Matrigel. The viral loads of EV-A71, CVB2, and PV-3 in cell lysate increased by about 3.8 log units, 4.0 log units, and 3.5 log units, respectively, at 72 hpi. The TCID_50_ assay showed that the infectious virus titer results were consistent with the viral load data. The virus titers of EV-A71, CVB2, and PV-3 plateaued at 48 hpi and were increased by about 2.5 log units, 4.0 log units, and 3.5 log units, respectively (Figure 2A). In contrast to these species A, B, and C enteroviruses, the viral load of EV-D68 did not increase significantly by 72 hpi even with a higher MOI of 1.0 (Figure 2B). In the immunofluorescence assay, abundant viral antigen expression and severe loss of structural integrity with cell lysis were observed in the organoids infected with EV-A71, CVB2, or PV-3, whereas mock-infected organoids demonstrated preserved integrity and absence of viral antigen expression (Figure 2C). Taken together, these results showed that human small intestinal organoids supported productive replication of EV-A71, CVB2, and PV-3, but not EV-D68.

### 3.3. mRNA Transcript Expression Profiles of Entry Receptors of EV-A71, CVB2, PV-3 and EV-D68 in RD Cells, Caco-2 Cells and Human Small Intestinal Organoids

**Figure 3:**
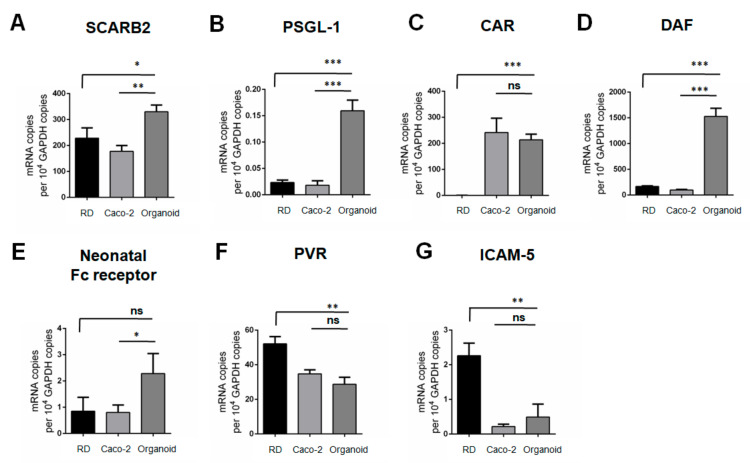
Intracellular mRNA transcript expressions of EV receptors in human small intestinal organoids. (**A**–**G**) Differential expressions of enterovirus receptors in rhabdomyosarcoma (RD) cells, Caco-2 cells, and small intestinal organoids. The expression levels were measured by quantitative PCR after cDNA synthesis. Data were normalized to 10^4^ GAPDH copies and shown as mean ± SD (in triplicates). * indicates *p* < 0.05; ** indicates *p* < 0.01; *** indicates *p* < 0.001; ns indicates not significant.

Based on the viral replication kinetics, we found that human small intestinal organoids supported productive replication of EV-A71, CVB2, and PV-3, but not EV-D68. To help explain the differential susceptibilities of human small intestinal organoids to these enteroviruses, we assessed the mRNA transcript expression levels of various enterovirus receptors in the organoids. We also compared the mRNA transcript expression levels of the receptors in the human small intestinal organoids with those in RD cells and Caco-2 cells which are commonly used cell lines for culturing enteroviruses. As shown in Figure 3A,B, the mRNA transcript expression levels of the EV-A71 receptors scavenger receptor class B member 2 (*SCARB2*) and P-selectin glycoprotein ligand-1 (*PSGL-1*) were significantly higher in the human small intestinal organoids than that in RD and Caco-2 cells. For CVB2, the mRNA transcript expression levels of the receptors decay-accelerating factor (*DAF*) and *neonatal Fc receptor* were also significantly higher in the organoids than in RD and Caco-2 cells, whereas the mRNA transcript expression level of coxsackievirus and adenovirus receptor (*CAR*) in the organoids was higher than that in RD cells and comparable to that in Caco-2 cells (Figure 3C–E). Additionally, the mRNA transcript expression level of the PV-3 receptor poliovirus receptor (*PVR*) in human small intestinal organoids was high (about 29 copies per 10^4^ GAPDH copies), and comparable with that of Caco-2 cells but lower than that of RD cells (Figure 3F). As for EV-D68 which did not replicate in the human small intestinal organoids, the mRNA transcript expression level of the receptor intercellular adhesion molecule 5 (*ICAM-5*) in the organoids was very low (0.49 copies per 10^4^ GAPDH copies) and significantly lower than that in RD cells which are permissive to EV-D68 infection (Figure 3G). Our data showed that human small intestinal organoids could express high levels of EV receptor mRNA transcripts that are required for productive replication of EV-A71, CVB2, and PV-3. The low *ICAM-5* mRNA transcript expression in the human small intestinal organoids may account for the organoids’ insusceptibility to EV-D68 infection. Overall, the receptor mRNA transcript expression levels corroborated with the viral replication kinetics and CPE patterns of species A to D enterovirus infections in human small intestinal organoids.

### 3.4. Comparative Perofrmances of Antiviral Assays in Human Small Intestinal Organoids and Cell Lines

**Figure 4:**
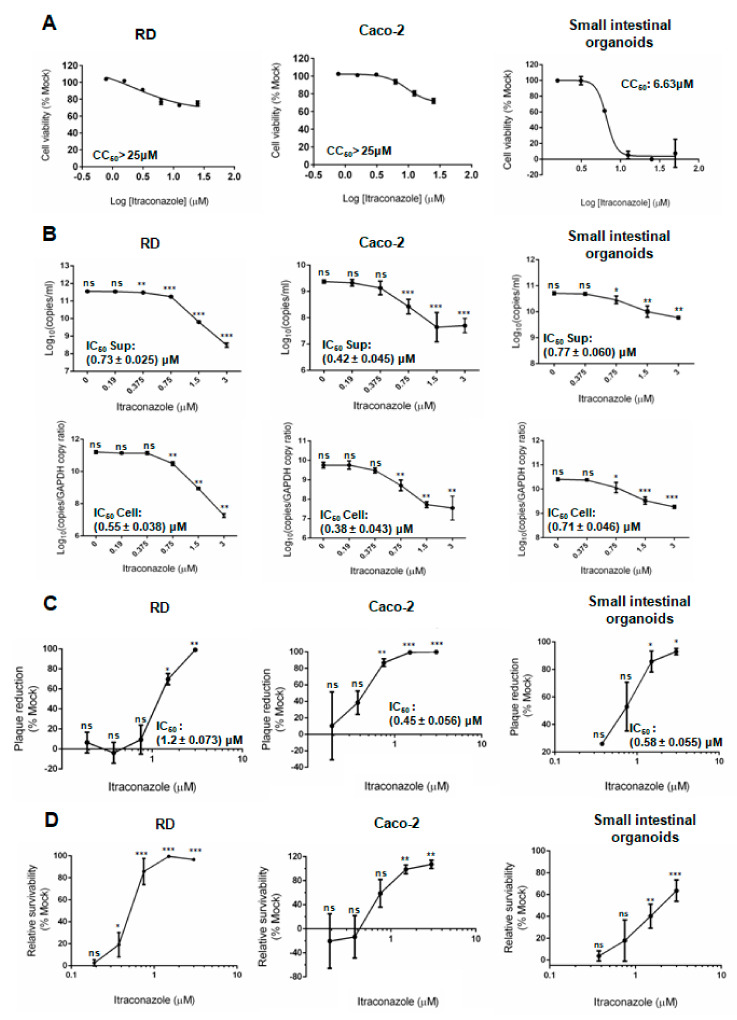
Cytotoxicity and antiviral activity of ITZ in RD cells, Caco-2 cells, and human small intestinal organoids. (**A**) Cell viability assay of ITZ in RD cells and Caco2 cells after 72 h of incubation and in organoids after 48 h of incubation. Abbreviations: CC_50_, 50% cellular cytotoxicity concentration; IC_50_, half maximal inhibitory concentration. (**B**) The inhibitory effect of ITZ on EV-A71 replication in RD cells, Caco-2 cells, and human small intestinal organoids by qRT-PCR. (**C**) The inhibitory effect of ITZ on EV-A71 infectious virus titer in RD cells, Caco-2 cells, and human small intestinal organoids by plaque reduction assay. (**D**) The cell protection effect of ITZ in EV-A71-infected RD cells, Caco-2 cells, and human small intestinal organoids. Data were shown as mean ± SD (in triplicates). * indicates *p* < 0.05; ** indicates *p* < 0.01; *** indicates *p* < 0.001; ns indicates not significant.

In order to evaluate the suitability of human small intestinal organoids as an antiviral evaluation platform, we tested ITZ in the organoids, RD cells, and Caco-2 cells, and compared ITZ’s cytotoxicity and antiviral activity in these cells. As shown in Figure 4A, the CC_50_ of ITZ in human small intestinal organoids (6.63 μM) was lower than that in RD cells and Caco-2 cells (>25 μM). In contrast, the IC_50_ of ITZ against EV-A71 was consistently determined to be <2 μM in human small intestinal organoids, RD cells, and Caco-2 cells in both the viral reduction assay and plaque reduction assay (Table 1). Dose-dependent reductions of EV-A71 viral load were observed in all three culture systems in the viral load reduction assay (Figure 4B). In the plaque reduction assay, ITZ treatment achieved complete or near complete plaque reduction in the organoids and cell lines (Figure 4C). Notably, the standard derivations of the IC_50_ obtained from the above assays in human small intestinal organoids were small, which suggested that this model has high reproducibility for antiviral evaluation. 

In the CPE inhibition assay, dose-dependent increases in the relative cell survivability were observed in the ITZ-treated human small intestinal organoids, RD cells, and Caco-2 cells. Importantly, we found that the relative survivability of ITZ-treated EV-A71-infected organoids (76.0%) was lower than that of ITZ-treated EV-A71-infected RD cells (96.7%), whereas Caco-2 cells showed very weak CPE and was therefore not a suitable model for the CPE inhibition assay (Figure 4D). Our results suggested that when compared with human small intestinal organoids, RD cells might overestimate the cell protection effects of potential antivirals and Caco-2 cells was not an optimal model for evaluating the cell protection effects for EV-A71 infection.

### 3.5. Immunofluorescence Analysis of ITZ Antiviral and Cell Protection Effects in EV-A71-Infected Human Small Intestinal Organoids

**Figure 5:**
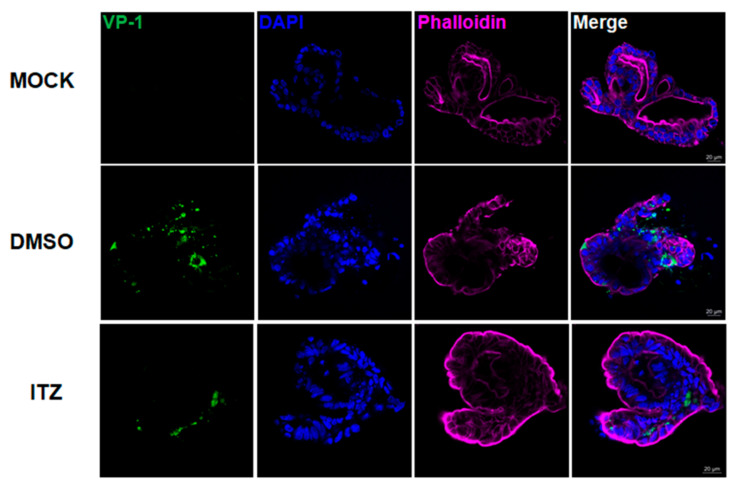
Immunofluorescence staining of EV-A71-infected human small intestinal organoids with or without ITZ treatment. Organoids with mock infection (upper panel), EV-A71 infection with DMSO control treatment (middle panel), and EV-A71 infection with 3 µM ITZ treatment (bottom panel), were immunostained for EV-A71 VP-1 antigen (in green). DAPI-stained nuclei were shown in blue. Phalloidin-stained cytoskeletons were shown in cyan and purple (scale bar: 20 μm).

The antiviral effect of ITZ treatment against EV-A71 in human small intestinal organoids was further evidenced by immunofluorescence staining. Abundant EV-A71 VP-1 antigen expression was found in most parts of the DMSO-treated organoids (Figure 5, middle panel). The DMSO-treated organoids exhibited perturbed structural integrity with leakage of intracellular contents. In contrast, we observed reduced EV-A71 VP-1 antigen expression in the ITZ-treated EV-A71-infected organoids (Figure 5, bottom panel). The structural integrity of the organoids was also preserved without leakage of intracellular content. These results showed that ITZ treatment effectively reduced viral replication and protected the host cells against EV-A71 infection.

### 3.6. Characterization of Host Response Changes after ITZ Treatment in EV-A71-Infected Human Small Intestinal Organoids

**Figure 6:**
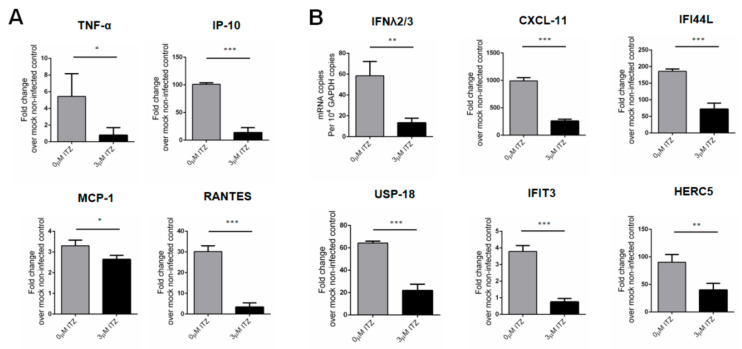
mRNA transcript expression profiles of the mediators involved in the host inflammatory and antiviral responses in EV-A71-infected human small intestinal organoids with or without ITZ treatment. (**A**) Intracellular mRNA expression of *TNF-α, IP-10*, *MCP-1,* and *RANTES* in EV-A71-infected organoids (MOI = 0.05) with or without 3 μM ITZ treatment at 48 hpi. (**B**) Intracellular mRNA expression of *IFNλ2/3*, *CXCL-11*, *IFI44L*, *USP-18*, *IFIT3*, and *HERC5* in EV-A71-infected organoids (MOI = 0.05) with or without 3 μM ITZ at 48 hpi. Data were shown as mean ± SD (in triplicates) and were normalized to mock-infected controls. * indicates *p* < 0.05; ** indicates *p* < 0.01; *** indicates *p* < 0.001.

Upon EV-A71 infection, the mRNA transcript expression levels of pro-inflammatory cytokines and chemokines, including tumor necrosis factor-α (*TNF-α*), interferon γ-induced protein 10 (*IP-10*), regulated on Activation, Normal T Cell Expressed and Secreted (*RANTES*), and monocyte chemoattractant protein-1 (*MCP-1*), were upregulated in the infected organoids. In addition, the infected organoids also induced expression of antiviral chemokines and interferon (IFN)-stimulated genes (ISGs) mRNA transcripts. Importantly, these pro-inflammatory cytokine/chemokine changes have also been reported to be associated with more severe diseases in EV-A71-infected patients, suggesting that the human small intestinal organoids represent a physiologically relevant model for characterization of the host immune response upon infection [33]. In contrast, after treatment with 3 μM ITZ, the mRNA transcript expression levels of type III interferon, ISGs, cytokines and chemokines that are involved in inflammatory and antiviral signaling were significantly reduced in the infected organoids (Figure 6A,B). These results indicated that the human small intestinal organoids generated a potent immune response upon EV-A71 infection and that ITZ treatment reduced these virus-induced host changes through inhibition of EV-A71 replication.

## 4. Discussion

In this study, we characterized the viral replication kinetics of representative virus strains from the four major human-pathogenic enterovirus species (i.e., EV-A: EV-A71, EV-B: CVB2, EV-C: PV-3, and EV-D: EV-D68) in human small intestinal organoids. We demonstrated that the human small intestinal organoids were permissive to infections by species A, B, and C enteroviruses and supported their productive replication, with the replication of CVB2 being the most efficient. In contrast, EV-D68 failed to induce observable CPE and its replication was poor in the human small intestinal organoids even using a higher virus inoculum (MOI = 1.0) than that of species A to C (MOI = 0.01) enteroviruses. To help explain these different viral replication kinetics of the enteroviruses, we further assessed the mRNA transcript expression levels of these enteroviruses’ receptors in the human small intestinal organoids. We showed that the organoids generally expressed relatively high mRNA transcript levels of receptors for EV-A71, CVB2, and PV-3, and low mRNA transcript level of the EV-D68 receptor *ICAM-5*. These findings are compatible with the clinical observation that EV-D68 primarily infects the nasopharyngeal cavity and the respiratory tract of humans [34] as it preferentially grows at 33 °C instead of 37 °C and is intolerable to the intestinal tract’s acidic conditions [35]. Our findings highlighted the potential of using human small intestinal organoids as a physiologically relevant model to investigate the pathogenesis of species A to C enteroviruses.

After establishing the viral replication kinetics of different enterovirus species in human small intestinal organoids, we next investigated whether this physiologically relevant system could be exploited as a novel antiviral evaluation platform for enterovirus infections. ITZ is an FDA-approved antifungal drug that has recently been shown to exhibit anti-EV-A71 activity in multiple cell lines such as RD rhabdomyosarcoma and BGM monkey kidney cells. We therefore used ITZ as a positive control to compare the performance of human small intestinal organoids with that of cell lines commonly used for in vitro evaluation of potential anti-EV-A71 drug compounds. Our results demonstrated that human small intestinal organoids could be readily used for determination of the IC_50_ and CC_50_ of ITZ using established antiviral assays as in RD and Caco-2 cells. Notably, while the IC_50_ of ITZ is similar among human small intestinal organoids, RD cells, and Caco-2 cells (0.58–1.20 μM), the CC_50_ was markedly lower in the organoids than RD and Caco-2 cells. Given that the human small intestinal organoids more closely resemble the in vivo state, our finding highlights the need to interpret cell toxicity in cancer cell lines in antiviral studies with caution as this would affect the consideration for further clinical development of potential antiviral agents. Moreover, the low CC_50_ of ITZ in human small intestinal organoids, together with the erratic serum drug concentrations achievable by oral dosing, shows our findings may suggest that intravenous rather than oral ITZ should be considered for clinical trials on patients with severe EV-A71 infection.

In contrast to cancer cell lines such as RD and Caco-2 cells, the human small intestinal organoids comprise non-transformed cells. Transformed cell lines are often defective in innate immune signaling which limits their use for studying the host immune response to viral infections [36]. For example, Caco-2 cells failed to demonstrate detectable innate immune responses, while human small intestinal organoids showed significant induction of cytokines and ISGs in response to astrovirus infection [37]. In this study, we demonstrated that human small intestinal organoids generated potent proinflammatory and antiviral responses upon EV-A71 infection which corroborated with clinical observations in severe EV-A71-infected patients. Importantly, we showed that ITZ treatment significantly reduced both the viral load and the proinflammatory response. This is of clinical relevance as a dysregulated systemic inflammatory response may contribute to brainstem encephalitis and non-cardiogenic pulmonary edema in severe EV-A71 infection [38,39].

Our study has some limitations. The spheroidal structure of the organoids may limit the access of virus to the organoids’ apical side, thus potentially reducing infection. To further improve this model, the 3D organoids may be dissociated into single cells and grown as a 2D monolayer culture so that the apical side of the organoids can be adequately exposed. Based on the promising results of ITZ against enterovirus infections in cell lines and in our human small intestinal organoid models, the drugs’ anti-enteroviral effects should be thoroughly evaluated in suitable animal models. Finally, to determine whether this human small intestinal organoids supports the replication of other enteroviruses, the viral replication kinetics of additional clinical and environmental virus isolates should be characterized in this model in the future [40,41]. 

Taken together, our results showed that the human small intestinal organoids model is a physiologically relevant model that supports productive replication of species A, B, and C enteroviruses but not EV-D68. We also demonstrated that this organoid model can serve as a reliable platform for evaluating anti-enteroviral agents. Furthermore, our findings provided novel data on ITZ’s antiviral effect and cytotoxicity in the human small intestines which may have implications on the optimal dosing of ITZ in patients under clinical trials.

## 5. Conclusions

Human 3D small intestinal organoids support productive replication of EV-A71, CVB2, and PV-3 but not EV-D68. This physiologically relevant culture model may serve as an important platform for studying the pathogenesis and antiviral treatment options of enteroviral infections. 

## Figures and Tables

**Table 1:** CC_50_, IC_50_, and selectivity indices of itraconazole (ITZ) in human small intestinal organoids, RD cells, and Caco-2 cells.

	CC_50_ (μM)	IC_50_ (μM)	Selectivity Index
Human small intestinal organoids	6.63	0.58 ± 0.055	11.43
RD	>25	1.20 ± 0.073	>20.83
Caco-2	>25	0.45 ± 0.056	>55.56

Selectivity Index = CC_50_/IC_50._ IC_50_ values were based on plaque reduction data and expressed as mean ± SD.

## Data Availability

The data that support the findings of this study are available from the corresponding author upon reasonable request.

## Appendix A

**Table A1:** Composition of human 3D small intestinal organoid culture medium.

Reagents	Suppliers	Working Concentration
Advanced DMEM–F-12 medium	Invitrogen	N/A
Penicillin-Streptomycin	Invitrogen	1%
HEPES buffer	Invitrogen	10 mM
GlutaMAX	Invitrogen	1X
Epidermal growth factor (EGF)	Invitrogen	50 ng/mL
Noggin-conditioned medium ^#^	N/A	10%
R-spondin-1-conditioned medium ^#^	N/A	20%
Wnt3A-conditioned medium ^#^	N/A	50%
Nicotinamide	Sigma-Aldrich	10 mM
Gastrin I	Sigma-Aldrich	10 nM
A-83-01	Tocris Bioscience	500 nM
SB202190	Sigma-Aldrich	10 μM
B27 supplement	Invitrogen	1X
N2 supplement	Invitrogen	1X
N-acetylcysteine	Sigma-Aldrich	1 mM

^#^ Conditioned media were produced from transfected cells which stably produce R-spondin-1, Noggin, and Wnt3A.

**Table A2:** A list of enteroviruses used in this study.

Species Group	Geographic Origin	Illness	GenBank Accession Number
EV-A71	Malaysia	Hand, foot and mouth disease	DQ341368.1
CVB2	Ohio	Flu-like symptoms	AF085363
PV-3	California	Fatal paralytic poliomyelitis	K01392
EV-D68	California	Lower respiratory illness	AY426531

**Table A3:** Primers for qRT-PCR/qPCR.

Genes	Primer Sequence	
PanEV	F	5′- GCCCCTGAATGCGGCTAAT -3′
	R	5′- ATTGTCACCATAAGCAGCCA -3′
	Probe	5′- 6FAM-CGGACACCCAAAGTAGTCGGTTCCG –lABkFQ -3′
hGAPDH	F	5′- ATTCCACCCATGGCAAATTC -3′
	R	5′- CGCTCCTGGAAGATGGTGAT -3′
SCARB2	F	5′- GCTGGGTGTGTTCTTTGGTTTG -3′
	R	5′- TTTCATCCGCTGTTCCCTCATC -3′
PSGL-1	F	5′- TGCTGCTCCTCTGACTT -3′
	R	5′- CCACACAGTCCCAAAGAA -3′
CAR	F	5′- TCATTCATCCCTGGGGTCCA -3′
	R	5′- TGAGGAGTGCGTTCAAAGTCT -3′
DAF	F	5′- TAAGGCTGTTTGAGGGATGC -3′
	R	5′- AATTCCTGGCGAGAAGGACT -3′
Neonatal Fc receptor	F	5′- AAGAAGAACCACAAGCGTTTTGA -3′
	R	5′- GACCCCAGTTCCTCTCTCACA -3′
PVR	F	5′- TGGACGGCAAGAATGTGACC -3′
	R	5′- ATCATAGCCAGAGATGGATACC -3′
ICAM-5	F	5′- CAAGCTCTGGTCACACTGGA -3′
	R	5′- GGGAGCGTATAGGACACGAA -3′
IP-10	F	5′- GAAATTATTCCTGCAAGCCAATTT -3′
	R	5′- TCACCCTTCTTTTTCATTGTAGCA -3′
MCP-1	F	5′- AGATCTGTGCTGACCCCAAG -3′
	R	5′- GGAGTTTGGGTTTGCTTGTCC -3′
RANTES	F	5′- ACCACACCCTGCTGCTTTGC -3′
	R	5′- CCGAACCCATTTCTTCTCTGG -3′
TNF-α	F	5′- GGCTCCAGGCGGTGCTTGTTC -3′
	R	5′- AGACGGCGATGCGGCTGATG -3′
IFNλ 2/3	F	5′- CCTGACGCTGAAGGTTCTGG -3′
	R	5′- ATATGGTGCAGGGTGTGAAGG -3′
CXCL11	F	5′- ATGAGTGTGAAGGGCATGGC -3′
	R	5′- TCACTGCTTTTACCCCAGGG -3′
IFI44L	F	5′- AACCTAGACGACATAAAGAGG -3′
	R	5′- CTGAAACCAAGTCTGCATAG -3′
USP18	F	5′- GACTCCTTGATTTGCGTTG -3′
	R	5′- TTGCTTGATAACTCCCTGG -3′
IFIT3	F	5′- TTCACCTGGAACTTATTCAAG -3′
	R	5′- TTTTATGTAGGCCAACAAGTT -3′
HERC5	F	5′- CAGAAAGTTGAATTTGTCGC -3′
	R	5′- CTGAGTCACTCTATACCCAAC -3′
